# Physical cell-cell contact elicits specific transcriptomic responses in wine yeast species

**DOI:** 10.1128/spectrum.00572-23

**Published:** 2024-07-16

**Authors:** Natasha A. Luyt, Riaan N. de Witt, Benoit Divol, Hugh G. Patterton, Mathabatha E. Setati, Patricia Taillandier, Florian F. Bauer

**Affiliations:** 1 Department of Viticulture and Oenology, South African Grape and Wine Research Institute, Stellenbosch University, Stellenbosch, Western Cape, South Africa; 2 Centre for Bioinformatics and Computational Biology, Stellenbosch University, Stellenbosch, Western Cape, South Africa; 3 Institut National Polytechnique de Toulouse, Paul Sabatier Université, Laboratoire de Génie Chimique, Université de Toulouse, Toulouse, France; University of Torino, Torino, Italy

**Keywords:** yeast transcriptomic responses, RNA-seq, yeast evolutionary adaptations, cell-cell interactions, wine ecosystem, microbial ecology

## Abstract

Fermenting grape juice provides a habitat for a well-mapped and evolutionarily relevant microbial ecosystem consisting of many natural or inoculated strains of yeasts and bacteria. The molecular nature of many of the ecological interactions within this ecosystem remains poorly understood, with the partial exception of interactions of a metabolic nature such as competition for nutrients and production of toxic metabolites/peptides. Data suggest that physical contact between species plays a significant role in the phenotypic outcome of interspecies interactions. However, the molecular nature of the mechanisms regulating these phenotypes remains unknown. Here, we present a transcriptomic analysis of physical versus metabolic contact between two wine relevant yeast species, *Saccharomyces cerevisiae* and *Lachancea thermotolerans*. The data show that these species respond to the physical presence of the other species. In *S. cerevisiae*, physical contact results in the upregulation of genes involved in maintaining cell wall integrity, cell wall structural components, and genes involved in the production of H_2_S. In *L. thermotolerans*, *HSP* stress response genes were the most significantly upregulated gene family. Both yeasts downregulated genes belonging to the *FLO* family, some of which play prominent roles in cellular adhesion. qPCR analysis indicates that the expression of some of these genes is regulated in a species-specific manner, suggesting that yeasts adjust gene expression to specific biotic challenges or interspecies interactions. These findings provide fundamental insights into yeast interactions and evolutionary adaptations of these species to the wine ecosystem.

**IMPORTANCE:**

Within the wine ecosystem, yeasts are the most relevant contributors to alcoholic fermentation and wine organoleptic characteristics. While some studies have described yeast-yeast interactions during alcoholic fermentation, such interactions remain ill-defined, and little is understood regarding the molecular mechanisms behind many of the phenotypes observed when two or more species are co-cultured. In particular, no study has investigated transcriptional regulation in response to physical interspecies cell-cell contact, as opposed to the generally better understood/characterized metabolic interactions. These data are of direct relevance to our understanding of microbial ecological interactions in general while also creating opportunities to improve ecosystem-based biotechnological applications such as wine fermentation. Furthermore, the presence of competitor species has rarely been considered an evolutionary biotic selection pressure. In this context, the data reveal novel gene functions. This, and further such analysis, is likely to significantly enlarge the genome annotation space.

## INTRODUCTION

The grape must environment is a complex ecosystem, and the interactions between microorganisms have been shown to influence the organoleptic properties of a wine. Interactions between *Saccharomyces cerevisiae* and non-*Saccharomyces* yeasts during wine fermentation have also been widely studied (1–4). These interactions largely define the development of the wine fermentation ecosystem during spontaneous fermentation (5). Interaction studies have also received significant impetus from the more recent development of multispecies inoculations. In the pursuit of wines with more unique sensorial characteristics, non-*Saccharomyces* yeasts indigenous to the grape must and vineyard environments have indeed emerged as a useful biotechnological resource (6–9). These include many yeasts belonging to genera such as *Pichia*, *Torulaspora*, *Lachancea*, *Hanseniaspora*, *Metschnikowia*, *Schizosaccharomyces,* and *Wickerhamomyces*, which, depending on fermentation parameters, can produce specific metabolites that contribute positively to wines (8). For instance, *Lachancea thermotolerans* in mixed fermentations with *S. cerevisiae* can increase glycerol content, overall acidity of wines, and enhance the production of certain favorable aromatic compounds (10–12). Similarly, *Torulaspora delbrueckii* can contribute to lower production of ethanol, certain short- and medium-chain fatty acids, and acetic acid, as well as higher production of esters and higher alcohols in wines (13, 14).

Data have revealed that physical cell-cell contact plays a significant role in regulating the ecological dynamics within this ecosystem (15–24). Depending on fermentation parameters, *S. cerevisiae* can have an inhibitory effect on certain non-*Saccharomyces* species in a cell-cell contact-dependent manner (15–24). The aforementioned interaction has been linked to the production of antimicrobial compounds (18) (linked to the accumulation of GAPDH-derived peptides on the cell-surface of *S. cerevisiae* when in co-culture with *L. thermotolerans*) (19). In other cases, metabolites secreted by *S. cerevisiae* seem to be the primary causative agent in the observed earlier death of *Hanseniaspora uvarum* during mixed fermentations (20). In contrast to these results, other authors implicated cell-cell contact with *S. cerevisiae* as the cause for an observed drop in viability in *H. uvarum* (23). Similar cell-cell mediated deaths in mixed culture with *S. cerevisiae* have also been observed for *T. delbrueckii* (17) and *Starmerella bacillaris* (21). For the study of such interactions, several types of compartmentalized bioreactors, which allow for two microbial populations to be separated physically while sharing the same growth medium, have been developed (17–24). This specialized equipment has been used to describe interactions between *S. cerevisiae* and several non-*Saccharomyces* spp. (such as *T. delbrueckii*, *L. thermotolerans*, *H. uvarum,* and *S. bacillaris*) and elucidate the impact of these interactions on fermentation dynamics and metabolite production (17–24). While such data highlight the consequences of physical vs metabolic contact on yeast performance and fermentation outcomes, the molecular mechanisms behind these interactions remain unclear, and to date, very few studies have focused on this aspect (25–29). Within the anthropogenic grape must fermentation ecosystem, the same dominant yeast species are observed globally, and it can be assumed that the presence of these competing cohabiting yeast species has resulted in specific evolutionary adaptations. While *S. cerevisiae* has been studied extensively in terms of its evolutionary adaptations to abiotic stresses, evolutionarily relevant responses to cohabiting species within this specific ecosystem have not been well described (30). Furthermore, the specific nature of the impact of physical, as opposed to metabolic contact, remains unknown.

To gain insight on the molecular mechanisms that are responsible for the impact of physical contact, the current study employed a previously described compartmentalized membrane bioreactor system (24) to elucidate transcriptional regulation within *S. cerevisiae* and *L. thermotolerans* in response to cell-cell and metabolic interactions. This experimental approach also allowed for further characterization of evolutionary adaptations within the yeast ecosystem. Genes of interest identified in the transcriptomic data set were further characterized for their expression at different stages of co-culture fermentations with *L. thermotolerans* and two other fermenting yeast species, *T. delbrueckii* and *Kluyveromyces marxianus*. These data suggest that physical contact between different species triggers both generic and species-specific responses.

## RESULTS

**Fig 1:**
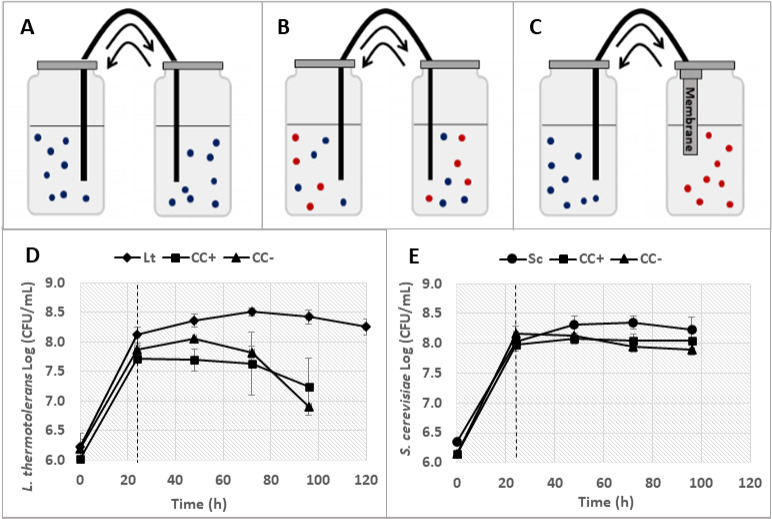
Images (A – C) illustrate fermentations conducted in a compartmentalized bioreactor. Arrows indicate the movement and mixing of media throughout fermentation. Blue and red dots represent two different yeast species. Fermentations were performed as monocultures of *L. thermotolerans* and *S. cerevisiae,* respectively, and image A illustrates these fermentations as the presence of a single species represented by only blue dots. Co-culture fermentations with cell-cell contact (CC+) were performed, and image B illustrates these fermentations as the presence of two species represented by blue and red dots in both compartments. Finally, co-culture fermentations without cell-cell contact (CC−) were performed with the inclusion of a membrane, and image C illustrates these fermentations as the presence of two species represented by red dots in one compartment and blue dots in the second compartment. Yeast growth was measured and represented as the log of viable colony counts (CFU/mL) for *L. thermotolerans* (**D**) and *S. cerevisiae* (**E**) within monoculture (Lt ♦; Sc ●), CC+ (■), and CC- (▲). Dashed lines indicate the 24 h RNA sampling points. Data are represented by two biological repeats. Error bars indicate standard deviation. Lt: *L. thermotolerans*; Sc: *S. cerevisiae*.

As described in the Materials and Methods section, the yeast were grown in a bioreactor system that kept two species physically separated while ensuring efficient exchange of the growth medium. The strains were grown in synthetic must under fermentative batch conditions in monoculture (Sc and Lt), co-culture with cell-cell contact (CC+), and in co-culture without cell-cell contact (CC−). Yeast growth was monitored throughout fermentation by sampling once a day and with the use of WL (Wallerstein) (Sigma-Aldrich, St. Louis, MO, USA) nutrient agar plates. Figure 1 illustrates each fermentation type and shows the growth of the two species in the three culture conditions expressed as colony forming units (CFU/mL). As previously described (24), physical contact had a significant impact on the growth rate of both species. *L. thermotolerans* reached a lower maximum viable cell count when it was in cell-cell contact with *S. cerevisiae* (CC+). When cell-cell contact was eliminated, the yeast was able to reach a higher cell density (CC−) (Fig. 1). The effect of the physical presence of a competitor was less pronounced in *S. cerevisiae*; however, a steady decline in cell counts can be observed for this yeast after 48 h until the end of fermentation in the absence of cell-cell contact (CC−).

### Transcriptome analysis

Samples for transcriptomic analysis by RNAseq were taken at 2 h and 24 h, and sequencing was performed as described in a previous study (28). Sample data were processed, differential gene expression analysis was performed using edgeR (31), and the different fermentations were compared (Table 1). Data with a log_2_ fold change of >1 and adjusted *P*-value of <0.05 were considered significant. The complete set of results of this analysis can be found in File S1.

**TABLE 1:** Fermentation settings that were compared, and interaction conditions under which differential gene expression was analyzed in *S. cerevisiae* (Sc) and *L. thermotolerans* (Lt)

	Factors influencing interaction in comparison	
Comparison	Cell-cell contact	Metabolic contact
CC+_VS_CC−	✓	X
CC+_VS_Sc/Lt	✓	✓
CC−_VS_Sc/Lt	X	✓

These data show that after 2 h, corresponding to early exponential phase of the cultures, few statistically significant differences in gene expression levels were observed between the cell-cell contact (CC+) and no-cell-cell contact co-cultures (CC−) (File S1). In contrast to the 2 h time point, many transcriptomic changes within the different comparisons were detected at the 24 h time point (Table 2). In *S. cerevisiae*, a similar amount of up- (217) and down-regulated (210) genes were detected when comparing the two co-culture fermentations (CC+_VS_CC−), whereas in *L. thermotolerans,* more genes were upregulated in this comparison (219 upregulated vs 139 downregulated genes). In the CC+_VS_Lt/Sc monoculture comparisons, the number of *L. thermotolerans* differentially expressed genes was more than double compared with *S. cerevisiae*. Overall, when comparing CC− with monocultures (CC−_VS_Lt/Sc), the least number of genes were differentially expressed, providing additional evidence that physical contact significantly impacts cellular responses in co-cultures (Table 2).

**TABLE 2:** Total differentially expressed genes in all comparisons for *S. cerevisiae* and *L. thermotolerans* at 24 h

	*S. cerevisiae*		*L. thermotolerans*	
	Upregulated	Downregulated	Upregulated	Downregulated
CC+_VS_CC−	217	210	219	139
CC+_VS_Sc/Lt	130	78	387	298
CC−_VS_Sc/Lt	167	99	65	71

**Fig 2:**
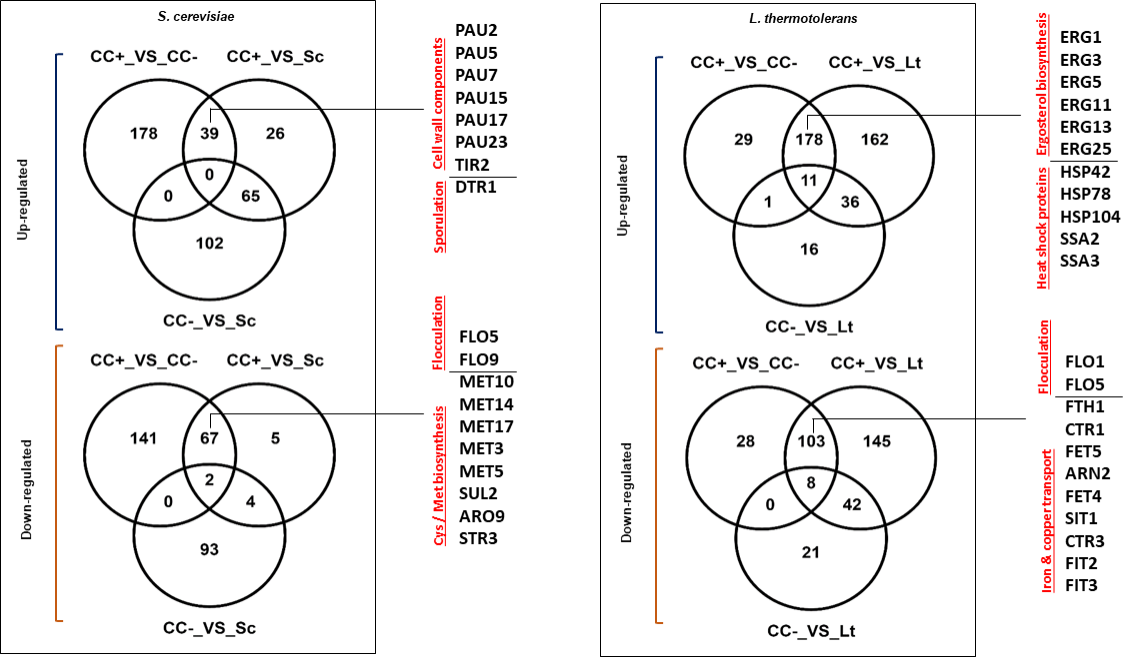
VENN diagrams illustrating common and unique genes that were either up- or down-regulated in *S. cerevisiae* and *L. thermotolerans* when comparing the monocultures (Sc; Lt) with the respective co-cultures (CC+; CC−) and when comparing the respective co-cultures. Some genes of interest shared between the CC+_VS_CC− and CC +VS_Lt/Sc comparisons are listed.

VENN diagrams (Fig. 2) were constructed to visually represent similarities in expression profiles of the respective yeasts in the different fermentation conditions/settings. When comparing the co-cultures (CC+_VS_CC−), it was observed that most of the 217 upregulated genes for *S. cerevisiae* were unique to this comparison. The small subset of upregulated genes, which were shared between the comparisons CC+_VS_CC− and CC+_VS_Sc (Fig. 2), is of particular interest regarding transcriptomic regulation as a result of cell-cell contact. The expectation would be that if a specific gene was highly regulated as a response to physical contact, it would be differentially expressed in both comparisons. Indeed, between these two comparisons, 39 upregulated genes were shared. More downregulated genes were shared (67 genes), with only a small number being unique to CC+_VS_Sc for *S. cerevisiae*.

Transcriptomic responses in *L. thermotolerans* were somewhat different to that of *S. cerevisiae*. Only 29 of the 219 upregulated genes in the CC+_VS_CC− comparison were unique, with 178 of these being shared with the CC+_VS_Lt comparison. Only 16 genes were unique to the CC−_VS_Lt comparison (which was substantially less than *S. cerevisiae*). A similar scenario unfolded for downregulated genes in this yeast.

### Gene ontology (GO) enrichment

A GO term enrichment analysis was performed to gain insights regarding the biological meaning behind the observed differentially expressed genes for *S. cerevisiae* and *L. thermotolerans*. In response to physical contact in the CC+_VS_CC− comparison, *S. cerevisiae* upregulated genes involved in cell wall organization or biogenesis or those forming structural components of the cell wall (Table 3). A considerable number of the genes encoding the cell wall integrity protein family *PAU* were upregulated (*PAU2*, *PAU5*, *PAU6*, *PAU7*, *PAU15*, *PAU17*, *PAU19*, and *PAU23*) as were several structural cell wall genes including *TIR1*, *TIR2*, *DAN1*, *DAN4,* and *PIR3*. Other upregulated genes in this comparison were related to spore formation (*DIT1*, *OSW2*, and *SPS22*) and cell wall biosynthesis (*KNH1* and *CHS3*), thiamine biosynthesis (*THI4*, *THI5*, *THI11*, *THI13*, *SNZ2*, *SNZ3*, and *THI12*), and genes coding for oxidoreductases involved in the Ehrlich pathway (*AAD4*, *AAD16*, *AAD14*, *ADH6*, and *ADH7*). Several genes related to allantoin catabolic processes were also upregulated (*DAL1*, *DAL2*, *DAL4*, *DAL7*, *DUR1,2*).

**TABLE 3:** GO terms enriched for biological processes (BP) in *S. cerevisiae* as a result of cell-cell contact in the CC+_VS_CC− comparison

	GO term	Log10 (*P*-value)	GO term description
	Upregulated		
*BP*	GO:0031505	−8.39	Fungal-type cell wall organization
	GO:0045229	−8.01	External encapsulating structure organization
	GO:0071554	−7.12	Cell wall organization or biogenesis
	GO:1901615	−4.03	Organic hydroxy compound metabolic process
	GO:1901617	−3.66	Organic hydroxy compound biosynthetic process
	GO:0042724	−2.76	Thiamine-containing compound biosynthetic process
	GO:0009228	−2.76	Thiamine biosynthetic process
	GO:0006081	−2.64	Cellular aldehyde metabolic process
	GO:0000256	−2.59	Allantoin catabolic process
	Downregulated		
*BP*	GO:0006811	−11.32	Ion transport
	GO:0034220	−8.82	Ion transmembrane transport
	GO:0010467	−5.76	Gene expression
	GO:0055085	−5.44	Transmembrane transport
	GO:1901678	−4.68	Iron coordination entity transport
	GO:0000097	−4.59	Sulfur amino acid biosynthetic process
	GO:0015891	−4.48	Siderophore transport
	GO:0046916	−4.17	Cellular transition metal ion homeostasis

Many of the enriched GO terms regarding downregulated genes were involved with iron, copper, and zinc transport (*FIT1*, *FIT2*, *FIT3*, *FET4*, *FRE2*, *FRE3*, *FRE4*, *FRE5*, *CTR3*, *ZRT1*, *ZRT2*, and *ZRT3*). Some amino acid transporters were downregulated (*TAT1*, *MMP1*, *AGP2*, *MUP1*, *MUP3*, *BAP2*, *BAP3*, *YCT1*, and *VBA3*). Under the enriched GO terms, “sulfur amino acid biosynthesis” and “metabolic processes” were genes involved in methionine and cysteine biosynthesis. These were downregulated, and many of these are involved in the sulfate reduction sequence (SRS) pathway (*SUL1*, *SUL2*, *MET3*, *MET14*, *MET16*, *MET5*, *MET10*, *MET2*, *MET17*, *CYS3*).

In *L. thermotolerans*, genes relating to ergosterol biosynthesis (*NCP1*, *HMG1*, *ERG1*, *ERG11*, *ERG13*, *ERG25*, *ERG3*, and ERG5) were enriched under the GO term “steroid metabolic process” (Table 4). Oxidation-reduction processes were upregulated, with 47 genes enriched to this GO term.

**TABLE 4:** GO terms enriched for biological processes (BP) in *L. thermotolerans* as a result of cell-cell contact in the CC+_VS_CC− comparison

	GO term no	Log10 (*P*-value)	GO term description
	Upregulated		
BP	GO:0055114	−9.12	Oxidation-reduction process
	GO:0022900	−6.23	Electron transport chain
	GO:0008202	−2.89	Steroid metabolic process
	GO:1902652	−2.31	Secondary alcohol metabolic process
	GO:0055085	−2.27	Transmembrane transport
	GO:0006091	−2.14	Generation of precursor metabolites and energy
	GO:0034220	−1.76	Ion transmembrane transport
	GO:0006811	−1.58	Ion transport
	Downregulated		
BP	GO:0000041	−7.45	Transition metal ion transport
	GO:0055076	−4.45	Transition metal ion homeostasis
	GO:0010467	−4.05	Gene expression
	GO:1903047	−3.19	Mitotic cell cycle process
	GO:0006811	−3.12	Ion transport
	GO:0051301	−2.90	Cell division
	GO:0015891	−2.88	Siderophore transport
	GO:0007049	−2.63	Cell cycle
	GO:1901678	−2.41	Iron coordination entity transport

Similar to *S. cerevisiae*, *L. thermotolerans* downregulated genes involved with iron, copper, and zinc transport (*FTR1*, *FTH1*, *FRE1*, *FRE3*, *FRE5*, *FET3*, *FET5*, *SIT1*, *CTR1*, *CTR3*, and *ZRT3*). These genes were enriched under most of the listed GO terms, such as “transition metal ion transport” and “siderophore transport” (Table 4).

### Consistent differential expression of genes as a result of cell-cell contact in *S. cerevisiae*

**Fig 3:**
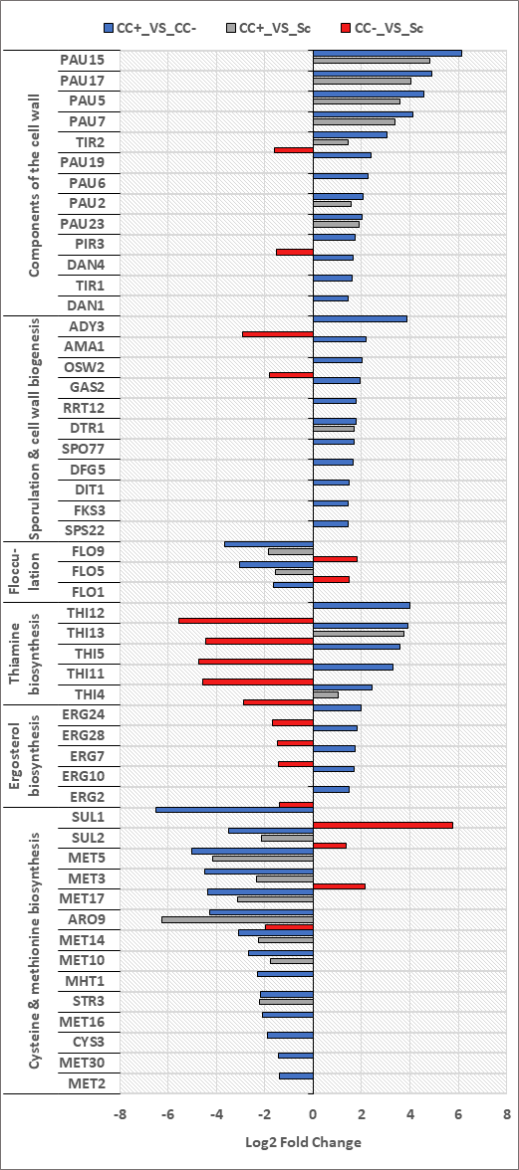
Differential expression of genes pertaining to specific biological processes or structural components of *S. cerevisiae* in the different fermentation comparisons (CC+_VS_CC−; CC+_VS_Sc; CC−_VS_Sc).

Genes consistently differentially expressed when comparing the two types of co-cultures (CC+_VS_CC−) and when comparing cell-cell contact co-cultures with monocultures (CC+_VS_Lt/Sc) were of particular interest. Common enriched GO terms between these two comparisons were identified and summarized in Fig. S1 and S2. Shared enriched GO terms were presented in relation to the percentage of enriched genes that make up the total genes associated with specific GO terms. Only GO terms with percentages higher than 10% were included in these figures. This analysis revealed a cluster of genes, which were consistently up- or down-regulated between these two comparisons. If the expression of these genes is related to cell-cell contact with another species, it is to be expected that contrasting differential expression would be observed between the comparisons with cell-cell contact and those without or that no significant change in expression would be observed in the comparisons without cell-cell contact. Indeed, for most of these genes, this is the case, and a summary of genes relating to the cell wall (in terms of structure and adhesion) and certain metabolites is summarized in Fig. 3. In *S. cerevisiae*, genes from the *PAU* gene family were upregulated consistently between these two comparisons (*PAU2*, *PAU5*, *PAU7*, *PAU15*, *PAU17,* and *PAU23*), and no differential expression was detected for these genes in the CC−_VS_Sc comparison (when cell-cell contact was not a contributing factor). Some of the highest log_2_ fold changes were also detected for these genes (ranging from 2 to 6). Other cell wall-related genes such as *TIR1*, *TIR2*, *DAN1*, *DAN4,* and *PIR3* were upregulated when comparing co-cultures (CC+_VS_CC−). *S. cerevisiae* upregulated genes related to spore formation and cell wall biogenesis in response to cell-cell contact, and for all but one of these genes (*DTR1*), no differential expression was detected in the CC+_VS_Sc comparison. A subset of genes involved in thiamine biosynthesis were upregulated when comparing the two co-cultures (*THI12*, *THI13*, *THI5*, *THI11*, and *THI4*), and ergosterol biosynthesis genes were upregulated in a similar manner. In the CC+_VS_CC− comparison, it was observed that *S. cerevisiae* downregulated genes relating to flocculation (*FLO1*, *FLO5*, and *FLO9*) and methionine and cysteine biosynthesis (*MET3*, *MET5*, *MET10*, *MET14*, *MET16*, *MET17*, and *SUL2*). For all but one gene (*SUL1*), these make up all genes encoding the pathway leading up to H_2_S formation. In contrast, these genes were upregulated at 2 h (File S1) when comparing the co-cultures (CC+_VS_CC−).

### Consistent differential expression of genes as a result of cell-cell contact in *L. thermotolerans*

**Fig 4:**
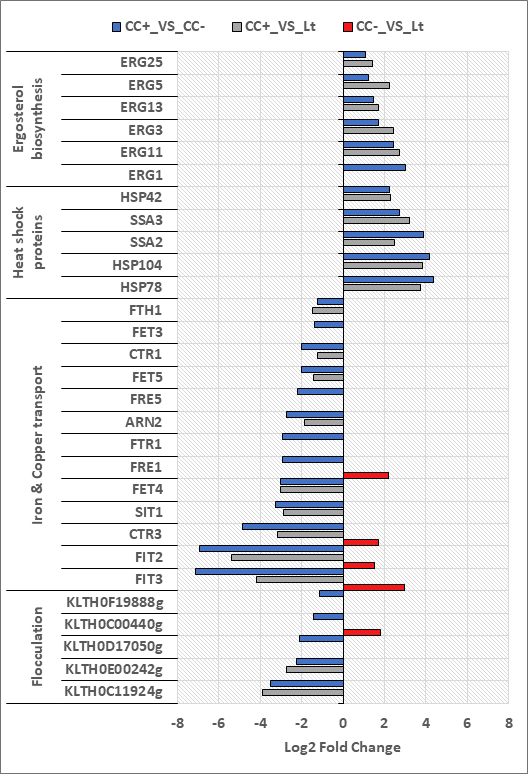
Differential expression of genes pertaining to specific biological processes of *L. thermotolerans* in the different fermentation comparisons (CC+_VS_CC−; CC+_VS_Lt; CC−_VS_Lt).

In *L. thermotolerans* (Fig. 4), genes involved in ergosterol biosynthesis were upregulated (*ERG1*, *ERG3*, *ERG5*, *ERG11*, *ERG13*, and *ERG25*) when comparing CC+ with CC−. All but one of these genes was upregulated when comparing CC+ with the monoculture. Heat shock protein (*HSP*) genes were upregulated in both cell-cell contact comparisons (*HSP42*, *HSP78*, *HSP104*, *SSA2*, *SSA3*).

Genes associated with iron and copper transport were downregulated when comparing CC+ with CC− (*FIT2*, *FIT3*, *FET4*, *FET3*, *FET5*, *FTR1*, *FRE1*, *FRE5*, *FTH1*, *SIT1*, *ARN2*, *CTR1*, and *CTR4*), with *FIT2* and *FIT3* log_2_ fold changes being some of the highest observed for *L. thermotolerans* (6.92 and 7.13, respectively). Similar to *S. cerevisiae* and when comparing the two co-cultures (CC+_VS_CC−), *L. thermotolerans* also downregulated genes involved with flocculation. These included *KLTH0E00242g*, *KLTH0D17050g*, *KLTH0C00440g*, *KLTH0F19888g* (which show similarity to *FLO1* in *S. cerevisiae*), and *KLTH0C11924g* (which shows similarity to *FLO5* in *S. cerevisiae*). Two of these were also downregulated when comparing CC+ with the monoculture (*KLTH0E00242g* and *KLTH0C11924g*), and all but one of these genes had no differential expression when comparing CC− with the monoculture (when cell-cell contact was not a contributing factor).

### qRT-PCR validation of transcriptomic data and further characterization of genes of interest

Following transcriptomic characterization of interactions between *S. cerevisiae* and *L. thermotolerans*, certain genes of interest were selected, and their roles in interaction were further characterized through qRT-PCR. The gene expression profiles generated for *S. cerevisiae* and *L. thermotolerans* when comparing CC+ with the respective monocultures also served as validation for the transcriptomic study. The genes of interest were further analyzed through relative gene expression determined at three different stages of fermentation and in co-culture with other relevant non-*Saccharomyces* yeasts (*T. delbrueckii* and *K. marxianus*).

**Fig 5:**
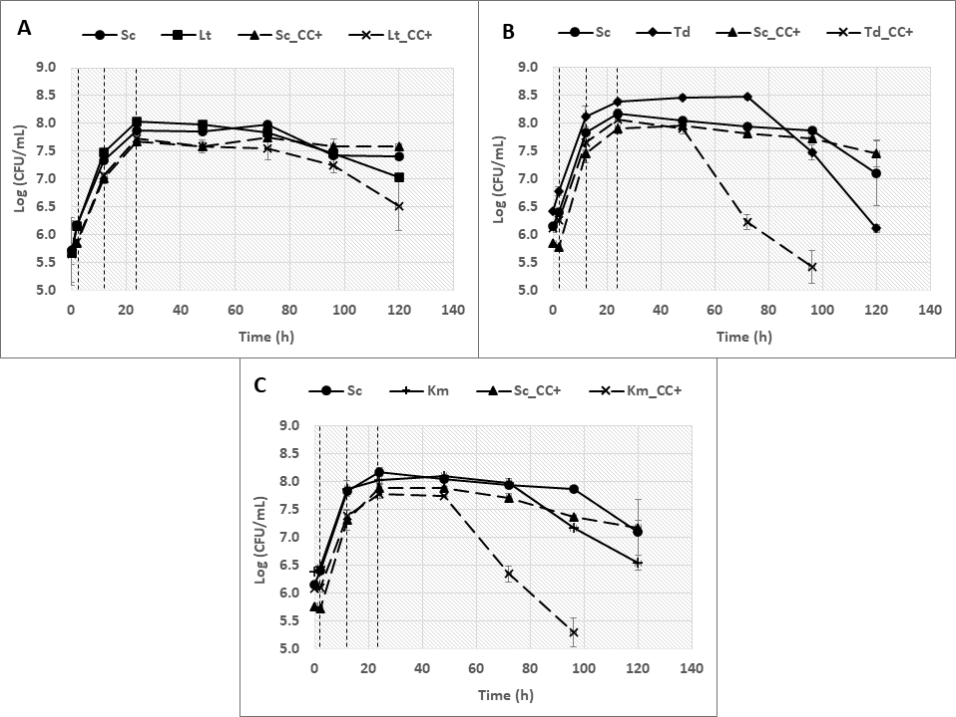
Yeast growth represented as the log of viable colony counts (CFU/mL) for each non-*Saccharomyces* pairing (*L. thermotolerans* [A], *T. delbrueckii* [B] and *K. marxianus* [C]) with *S. cerevisiae*. Monoculture data (Sc ●; Lt ■; Td ♦; Km **+**) are represented as solid lines, and co-culture with physical contact (Sc ▲; non-*Saccharomyces* yeast **X**) is represented as dashed lines. Vertical dashed lines indicate the 2 h, 12 h, and 24 h RNA sampling points. Data represented by three biological repeats. Error bars indicate standard deviation. Sc: *S. cerevisiae*; Lt: *L. thermotolerans*; Td: *T. delbrueckii*; Km: *K. marxianus*.

In co-culture, growth as a measure of viable colony counts for *S. cerevisiae* and *L. thermotolerans* (Fig. 5A) was similar to results from fermentations in the compartmentalized bioreactor. The two yeasts achieved high cell densities in monocultures and slightly lower when in co-culture. In the latter fermentations, *S. cerevisiae* was able to persist until the end. This contrasts with *L. thermotolerans*, in which cell concentrations gradually declined from 48 h. Nevertheless, viable *L. thermotolerans* cells were still present at high densities by the end of fermentation. Similarly, when grown in co-culture with *T. delbrueckii*, *S. cerevisiae* viable cell counts were slightly lower compared with its monoculture, and although the counts were slowly declining, the yeast was still able to persist until the end of fermentation (Fig. 5B). In contrast, *T. delbrueckii* viable cell counts were significantly lower compared with its monoculture. This yeast initially achieved higher cell numbers compared with *S. cerevisiae,* and in contrast to *L. thermotolerans*, cell numbers rapidly declined from 48 h. After 96 h, no viable cells were detected. A similar scenario unfolded for *K. marxianus* in co-culture with *S. cerevisiae* (Fig. 5C). In monoculture, growth for the two yeasts was comparable, with a decline in cell numbers occurring slightly earlier for *K. marxianus* and both yeasts persisting until the end of fermentation. The yeasts had a similar growth pattern in co-culture until 48 h. From this time point, *K. marxianus* growth declined significantly and no viable cells were detected after 96 h.

Relative gene expression levels were quantified in monoculture (control) and co-culture with cell-cell contact (experimental) and expressed as log_2_ fold changes. These were then compared between time points (stages of fermentation) and between the different non-*Saccharomyces* pairings.

### Comparison of gene expression during different stages of fermentation

**Fig 6:**
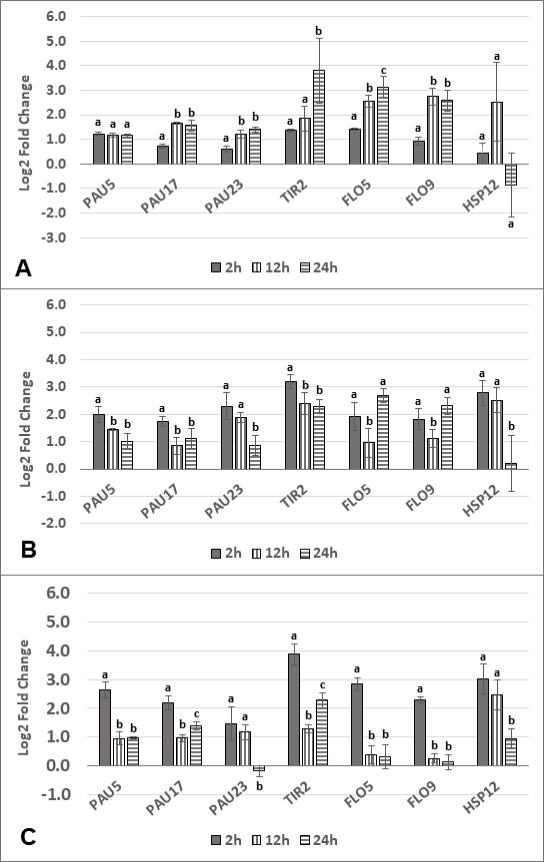
Relative gene expression represented as log_2_ fold changes for a subset of *S. cerevisiae* genes at different time points (2 h, 12 h, and 24 h) in co-culture with *L. thermotolerans* (**A**), *T. delbrueckii* (**B**), and *K. marxianus* (**C**). For each gene, statistical differences were calculated between the three-time points. Statistical significance is indicated with lowercase letters by a *P*-value threshold of <0,05. Statistically similar values are represented by the same letter, and statistically different values are represented by different letters. Error bars indicate standard deviation.

*S. cerevisiae* gene expression was quantified at three-time points in the co-cultures with the three different yeast species: entry into exponential growth phase (2 h), mid-exponential phase (12 h), and late exponential phase (24 h) (Fig. 6). Except for *FLO* genes, the results (*PAU* and *TIR* genes) correlate well with the transcriptomic study, which had been carried out at the 24 h time point. In co-culture with *L. thermotolerans* (Fig. 6A), the expression of most genes was higher at the later time points compared with 2 h (*PAU17*, *PAU23*, *TIR2*, *FLO5*, and *FLO9*). While *FLO5* and *FLO9* were upregulated in this experiment, this result stands in contrast to the transcriptomic analysis in bioreactors at 24 h. *PAU5* expression appeared to be relatively consistent between all stages of fermentation, and no clear trend could be observed for *HSP12*, with high standard deviation between biological repeats. This was also observed for *S. cerevisiae HSP12* expression in co-culture with *T. delbrueckii* (Fig. 6B). Here, *PAU* expression was usually higher at the earlier time points compared with 24 h. A similar trend was observed for *TIR2* and *HSP12. FLO5* and *FLO9* expressions were nearly identical, with similar expression levels at early and late exponential phase (2 h and 24 h) and lowest being at mid-exponential phase (12 h). In fact, trends for *FLO* gene expression were different between all three non-*Saccharomyces* pairings. Like *T. delbrueckii, S. cerevisiae PAU*, *TIR2,* and *HSP12* gene expression was similar in co-culture with *K. marxianus* (Fig. 6C), whereas *FLO5* and *FLO9* expression was the highest early in fermentation.

**Fig 7:**
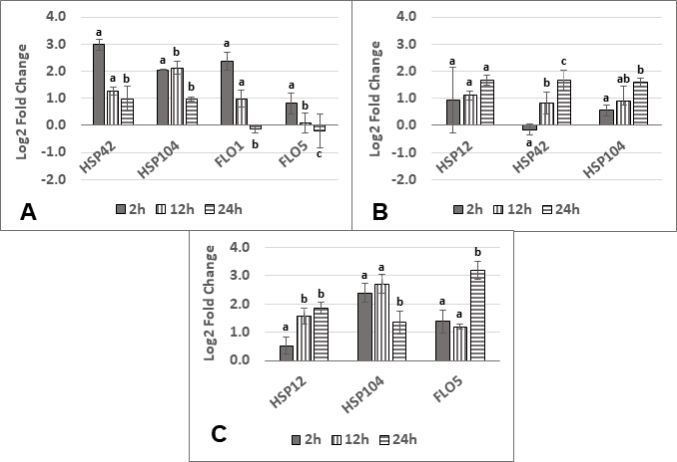
Relative gene expression represented as log_2_ fold changes for a subset of genes at different time points (2 h, 12 h, and 24 h) belonging to *L. thermotolerans* (**A**), *T. delbrueckii* (**B**), and *K. marxianus* (**C**) in co-culture with *S. cerevisiae*. For each gene, statistical differences were calculated between the three-time points. Statistical significance is indicated with lowercase letters by a *P*-value threshold of <0,05. Statistically similar values are represented by the same letter, and statistically different values are represented by different letters. Error bars indicate standard deviation.

The expression of some non-*Saccharomyces* genes of interest was also quantified (Fig. 7). In co-culture with *S. cerevisiae*, the general trend for all genes tested (*HSP42*, *HSP104*, *FLO1,* and *FLO5*) in *L. thermotolerans* was a higher expression early in fermentation compared with 24 h. In co-culture with *S. cerevisiae*, *T. delbrueckii HSP12* expression exhibited high standard deviation at 2 h and was consistently upregulated at mid- to late-exponential phase (Fig. 7B). For this yeast, *HSP42* and *HSP104* expression was highest later in fermentation (which is in contrast to *L. thermotolerans*). In *K. marxianus* (Fig. 7C), *HSP12* expression was highest at the later time points while the opposite was observed for *HSP104*. In contrast to *L. thermotolerans, FLO5* was upregulated in co-culture at all time points with the highest relative gene expression occurring at 24 h.

### Comparison of *S. cerevisiae* relative gene expression between different non-*Saccharomyces* pairings

**Fig 8:**
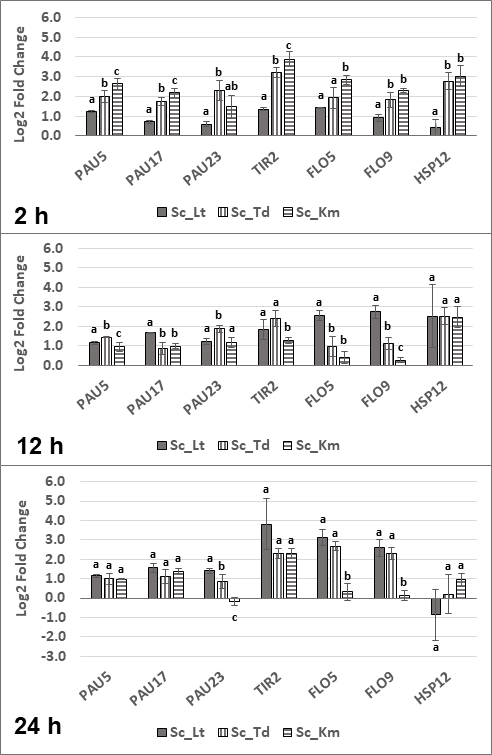
Relative gene expression represented as log_2_ fold changes for a subset of *S. cerevisiae* (Sc) genes at different time points (2 h, 12 h, and 24 h) in co-culture with *L. thermotolerans* (Sc_Lt), *T. delbrueckii* (Sc_Td), and *K. marxianus* (Sc_Km). For each gene, statistical differences were calculated between the three non-*Saccharomyces* pairings. Statistical significance is indicated with lowercase letters by a *P*-value threshold of <0,05. Statistically similar values are represented by the same letter, and statistically different values are represented by different letters. Error bars indicate standard deviation. Sc: *S. cerevisiae*; Lt: *L. thermotolerans*; Td: *T. delbrueckii*; Km: *K. marxianus*.

Relative gene expression profiles were also compared between species at each time point (Fig. 8). At the early exponential phase (2 h), all evaluated *S. cerevisiae* genes were upregulated in co-culture with all three non-*Saccharomyces* yeasts. Broadly, expression of genes was highest in *K. marxianus* compared with the other two non-*Saccharomyces* yeasts. At mid-exponential phase (Fig. 8, 12 h), there were minor differences between *PAU* gene expression. Similar expression levels were obtained for *TIR2* in *T. delbrueckii* and *L. thermotolerans* pairings and were also higher compared with *K. marxianus. FLO5* and *FLO9* were expressed significantly more in co-culture with *L. thermotolerans* compared with the other two non-*Saccharomyces* yeasts. Relative gene expression of *S. cerevisiae* genes was the most similar between non-*Saccharomyces* yeasts at the late exponential phase (Fig. 8, 24 h). Here, most of the genes were upregulated, and there were no statistical differences between the different non-*Saccharomyces* pairings for *PAU5*, *PAU17*, *TIR2,* and *HSP12* log_2_ fold changes. Similar expression levels were observed for *FLO5* and *FLO9* in co-culture with *L. thermotolerans* and *T. delbrueckii,* and these were significantly higher than *K. marxianus*.

## DISCUSSION

In the present study, the previously reported antagonistic relationship between *S. cerevisiae* and *L. thermotolerans* (24) was explored further. The transcriptomic analysis revealed a larger number of differentially expressed genes for *S. cerevisiae* and *L. thermotolerans* at 24 h versus 2 h in the different comparisons. This might be due to the fermentation medium still being abundant in nutrients at 2 h; therefore, competition for these was limited. At this time point, cells were also still adjusting to the new environmental conditions and consequently were in lag phase. Cell concentrations were also considerably lower; therefore, less cell-cell contact was occurring versus at 24 h.

At 24 h and in *S. cerevisiae*, fewer differentially expressed genes were shared between the cell-cell contact comparisons compared with *L. thermotolerans* (Table 2). *S. cerevisiae* persisted relatively consistently within the respective fermentations and was not as affected as *L. thermotolerans* by the physical presence of another yeast. This likely explains the greater transcriptomic response in *L. thermotolerans*. This transcriptomic regulation in *L. thermotolerans* aligns well with previously reported co-culture studies of *S. cerevisiae* and *L. thermotolerans* (identical strain) under anaerobic conditions in a similar fermentation medium but using very different, semi-continuous fermentation conditions (28).

In the transcriptomic analysis, iron and copper uptake were downregulated in both *S. cerevisiae* and *L. thermotolerans. FIT2* and *FIT3* are cell wall genes responsible for retaining siderophore bound iron in the cell wall before being taken up into the cell (32). Their expression is also induced by iron deprivation. The higher expression of these genes in CC− could be explained by the ability of *L. thermotolerans* to survive better when not in physical contact with *S. cerevisiae*. Therefore, iron availability could have been lower, resulting in earlier deprivation in CC−, which could have caused the observed increased regulation of iron uptake in both yeasts. The downregulation of iron and copper uptake genes has been reported before (28) and in particular for *L. thermotolerans* with *FIT2* and *FIT3* log_2_ fold changes similar to the current study. In response to physical contact, *S. cerevisiae* also upregulated genes involved with thiamine biosynthesis. This process is upregulated in response to low thiamine levels (33), and the compound is essential in the production of thiamine-pyrophosphate, which serves as a cofactor for pyruvate decarboxylase.

Similar to the present study, two thiamine biosynthesis genes (*THI11* and *THI13*) have previously been reported to be upregulated by *S. cerevisiae* in co-culture with *L. thermotolerans* (29). In a mixed culture of *S. cerevisiae* and *Hanseniaspora guilliermondii*, the increased expression of two thiamine biosynthesis genes (*THI20* and *THI21*) was also observed (25). Previous reports have noted the absence of thiamine biosynthesis genes in *Hanseniaspora* species (34) and that a subset of *Lachancea* species only contained a single copy or none of the *THI5* gene family (35). The absence of these genes in the non-*Saccharomyces* species likely led to an increase in competition for thiamine and in doing so, forced *S. cerevisiae* to synthesize it *de novo*.

Ergosterol biosynthesis genes were upregulated in response to cell-cell contact in both yeasts. This sterol is incorporated into the fungal cell membrane where it plays a role in stabilizing membrane structure, regulating fluidity, permeability, and membrane-bound enzyme activity (36) and has been shown to play an important role in stress adaptation during alcoholic fermentation (37, 38).

The SRS pathway is not only induced when there is a metabolic demand for cysteine and methionine in *S. cerevisiae* (39) but seemingly by a range of factors such as higher expression of thiamine biosynthesis genes (40) and a lack of vitamins such as pantothenate and vitamin B6 (41, 42). In response to cell-cell contact, genes encoding the complete pathway leading up to the point of H_2_S formation as well as *MET17* and *CYS3* (cysteine and methionine biosynthesis) were downregulated at 24 h, whereas all but one of these genes (*CYS3*) were upregulated at 2 h. Since H_2_S concentrations were not monitored, it is difficult to speculate whether the increased expression of these genes in CC+ was solely aimed at H_2_S production, a dual action of H_2_S production and amino acid synthesis or purely the latter. A previous study (43) demonstrated that overexpression of *MET17* in *S. cerevisiae* led to either unchanged or decreased levels of H_2_S, but at the same time, the resulting increased enzymatic activity did not lead to an increase in cysteine production either. However, these authors do not account for the fact that released H_2_S can react with other compounds present in the fermentation, which might explain why no differences or decreased values were observed (39). Nitrogen deficiency early in fermentation (2 h) is an unlikely scenario. Given the fact that there would not have been a strong metabolic demand for cysteine or methionine synthesis at this time point, the need for *S. cerevisiae* to express these genes more at 2 h (when in physical contact with *L. thermotolerans*) indicates a possible role in cell-cell interaction. Indeed, the idea that H_2_S production may offer *S. cerevisiae* a competitive advantage over cohabiting yeasts by inhibiting respiration and oxidative metabolism has been hypothesized before (39, 44) but has not been verified. This hypothesis merits further investigation.

In response to cell-cell contact, *S. cerevisiae* responded by upregulating structural elements of the cell wall and genes involved in maintaining cell wall integrity. In particular, the *PAU* genes seemed important in this strategy and their expression in *S. cerevisiae* has been shown to be induced by the stressful environment during wine fermentation (45–47). These genes have also been linked to yeast interactions (27, 28). The fact that no differential expression for *PAU* genes was detected when physical contact was excluded indicates that their expression is induced by the physical presence of a cohabiting yeast through a cell-cell trigger and could also partly explain why *S. cerevisiae* experienced a slight drop in culturability in the CC− fermentations. These results were validated in the qRT-PCR approach in co-culture with *L. thermotolerans*. In this experiment, the expression of *PAU* genes was consistently upregulated and higher at 24 h for two *PAU* genes. In contrast to co-fermentations with *L. thermotolerans*, *PAU* gene expression was, in most cases, higher at the earlier time points in co-culture with both *T. delbrueckii* and *K. marxianus*. These results broadly align with those of a previous study (27). *S. cerevisiae*’s expression of *PAU* genes in co-culture seems to be vital at the onset of fermentation and appears to be modulated differently depending on interacting species.

Other *S. cerevisiae* cell wall genes, which were upregulated in the transcriptomic cell-cell contact comparisons, were *TIR1*, *DAN1*, *DAN4*, *PIR3,* and *TIR2*. The expression of genes belonging to the *DAN/TIR* family is triggered by anaerobiosis (48–50) and some by cold shock (51, 52). These genes encode cell wall mannoproteins and play a role in cell wall permeability (53). In *S. cerevisiae, TIR2* was upregulated at 24 h in co-culture with *L. thermotolerans* and, therefore, further validated the transcriptomic results. The differential expression of genes belonging to the *DAN/TIR* family in response to biotic stresses in co-culture with *T. delbrueckii* and *K. marxianus* has not been reported before.

Another cell wall-related response was the consistent downregulation of flocculation genes in the transcriptomes of both *S. cerevisiae* (*FLO5* and *FLO9*) and *L. thermotolerans* (*FLO1* and *FLO5*) in response to cell-cell contact. Similarly, *FLO1* was previously found to be downregulated in *L. thermotolerans* in co-culture with *S. cerevisiae* (28). While *FLO* genes have been studied extensively in *S. cerevisiae* (54, 55), their exact role in relation to interaction and adaptation to cohabiting yeasts within a wine ecosystem has not been well documented. Co-adhesion between different species has recently been reported (56, 57), and these authors proposed a supplementary role of *FLO* gene expression as a way for cohabiting yeasts to control ecosystem dynamics. The fact that *FLO* gene expression was downregulated in response to cell-cell contact suggests a strategic response whereby these species avoid co-adhesion and, therefore, not to offer an adaptive advantage to the cohabiting species. At 24 h, qRT-PCR *FLO* expression data generated for the yeast pair *S. cerevisiae–L. thermotolerans* did not correlate with the transcriptomic data. The regulation of *FLO* genes and factors contributing to their expression remains elusive and is perpetuated by often contradicting and varying *FLO* gene expression profiles (28, 58). Such variability is likely due to differences in experimental layout, and the fact that genes located in sub-telomeric regions have been proposed to be under epigenetic regulation to hedge for changing environmental conditions (54, 59). Such epigenetic changes could serve as a strategy for *S. cerevisiae* to not commit all of its cells to one phenotype (60) and, in this way, modulate population dynamics in different ways in different parts of the fermentation. While the RNA-seq and qPCR data represent a population average, a small percentage of individual cells may be responding differently, expressing *FLO* genes at higher or lower levels than average. Such a survival strategy would also aid parts of the population to be pre-adjusted to future environmental changes. When comparing qRT-PCR with RNA-seq, a high concordance between these two methods was observed, with only 15%–20% of genes tested showing contrasting expression profiles, regardless of RNA-seq workflow chosen (61). Most of these “non-concordant” genes had differential expression levels < 2, and some were found to be expressed at very low levels. In our RNA-seq and validation approach, *FLO* genes possibly fall into the “non-concordant” category, and future work would have to consider different validation approaches for these genes. Regardless of these results, RNA-seq is still considered the standard for these types of transcription studies (61), and the results obtained in the present data set are of great value to our understanding of yeast interactions and gene functions. *S. cerevisiae FLO* gene expression was consistently different between species at each time point in the qRT-PCR approach. The most notable difference can be observed at late exponential phase. Here, expression for most of the other genes was statistically similar, whereas *FLO* gene expression appeared to be species-dependent. *S. cerevisiae FLO5* and *FLO9* expressions were similar between the co-culture fermentations with *L. thermotolerans* and *T. delbrueckii,* and these were significantly higher than *K. marxianus*. Since *L. thermotolerans* and *T. delbrueckii* are more common cohabiting yeasts of grape must than *K. marxianus*, this observation could indicate a possible strategy evolved over time aimed at specific species by *S. cerevisiae*. When *S. cerevisiae FLO* gene expression in co-culture was at its highest, the expression of *FLO1* and *FLO5* in *L. thermotolerans* and *K. marxianus,* respectively, at the corresponding time point, was at its lowest. This again points to an avoidance of co-adhesion by all species involved. These data correlate well to a previous report that co-adhesion of a *S. cerevisiae* overexpressing *FLO5* strain with *L. thermotolerans* led to a loss in viability in *S. cerevisiae* (57). This serves as motive behind the downregulation of *FLO* genes in response to cell-cell contact in *S. cerevisiae*: to avoid a loss in viability. In the same study, the viability in *L. thermotolerans* was increased. Thus, in the current study, the latter might be regulated in *L. thermotolerans*/*K. marxianus* to avoid adhesion with *S. cerevisiae* (57).

Previous studies have linked the stress-inducible heat shock protein (*HSP*) genes to yeast interactions. Several *HSP* genes were upregulated in *L. thermotolerans* in response to cell-cell contact (such as *HSP42* and *HSP104*). Upregulation of these genes has been observed in both *S. cerevisiae* and *T. delbrueckii* in co-culture (27). In *S. cerevisiae*, *HSP104* affords tolerance to heat, ethanol, and arsenate (62) by acting as a protein disaggregation agent (63), whereas *HSP42* acts as a sorting label by co-aggregating with misfolded proteins generated from heat shock (64). These roles have not been assigned in *L. thermotolerans*; however, these data suggest an additional role of *HSP* proteins as a stress response to a cohabiting yeast within a specific ecosystem. It has been suggested that *HSP12* may be released into the extracellular environment to act as a stress signal and that the combined action of the Pau5p, Hsp12p, and killer toxins offers *S. cerevisiae* a way to regulate production and utilization of nutrients within the ecosystem, even if it may come at a cost to itself (65). The expression of this gene in *S. cerevisiae* was not consistent in co-culture with *L. thermotolerans*, with much variation observed between replicates. Differential expression of this gene was high at 2 h when in co-culture with *T. delbrueckii* and *K. marxianus,* respectively. In both cases, gene expression was significantly lower at 24 h. The data correlate to a previous study that observed that *S. cerevisiae* upregulated *HSP12* expression at 2 h and 12 h in co-culture with *T. delbrueckii* (27). Taking the current data set into account and since these authors also observed upregulation of *PAU5* at 2 h, the combined action of these two genes might not only be a strategy utilized between *S. cerevisiae* strains (65) but also aimed at different non-*Saccharomyces* species, in a possible species-specific manner. The observed drop in viable cell counts for *T. delbrueckii* and *K. marxianus* after 48 h might also be linked to this combined action. However, this observation, as well as the apparent species-specific *PAU, TIR2,* and *HSP12* responses, requires further investigation. In co-culture with all three non-*Saccharomyces* yeasts, *S. cerevisiae HSP12* was upregulated at 24 h at high log_2_ fold changes, and its expression in co-culture was highest at this time point when compared with single culture. This trend was similar for *HSP42 and HSP104* in *T. delbrueckii* and confirms previous results (27). *HSP104* expression in *L. thermotolerans* and *K. marxianus* was similar, and since these two non-*Saccharomyces* yeasts are closely related (66), these results may indicate a conserved role in interactions for *HSP104* between *L. thermotolerans* and *K. marxianus*, but again, this needs further investigation.

### Conclusions

In the current study, the molecular responses to physical interspecies contact between *S. cerevisiae* and *L. thermotolerans* were explored. The data provide evidence that different yeast species exhibit different adaptive strategies. *S. cerevisiae* responses were cell wall focused and included the higher expression of genes involved with maintaining cell wall integrity and structure of the cell wall (*PAU*, *DAN*, and *TIR*). The early induced expression of SRS-pathway genes by *S. cerevisiae* might also be linked to a strategy aimed at outcompeting a cohabiting yeast, but further investigation is needed to confirm this. In turn, *L. thermotolerans* responded by increasing the production of stress response genes (*HSP*), whereas both yeasts avoided co-aggregation during co-fermentation.

Beyond these adaptations, the data provide the first evidence for species-specific interaction responses since differential transcriptomic responses as a result of cell-cell contact between *S. cerevisiae* and non-*Saccharomyces* yeasts have not been reported before. Species specific differential expression was observed for *PAU*, *TIR2*, *HSP12,* and *FLO* genes in *S. cerevisiae*. The species-specific regulation of adhesion genes occurred between two closely related non-*Saccharomyces* yeasts, and avoidance of co-adhesion appeared to be a response. Taken together with the data from Conacher et al. (29), the data provide a novel perspective on fundamental molecular mechanisms that may regulate ecosystem functioning and reveal novel functional roles of *S. cerevisiae* and non-*Saccharomyces* genes.

The data provide an important baseline for future yeast ecosystem and interaction studies. They also provide new avenues for the investigation of the role and mechanisms of biotic selection pressures in the evolutionary adaptations of yeast species to ecological niches.

## MATERIALS AND METHODS

### Fermentations in a compartmentalized bioreactor system

Synthetic grape must monoculture and co-culture fermentations (Table 5) were performed using the commercial *S. cerevisiae* strain Lalvin EC1118 (Lallemand Inc., Montreal, QC, Canada) and *L. thermotolerans* IWBT Y1240 (CBS 16374). The latter yeast was obtained from the South African Grape and Wine Research Institute (SAGWRI) yeast culture collection at Stellenbosch University, South Africa. Medium composition, inoculation strategies, and fermentation parameters were done according to previous compartmentalized fermentations (24). Fermentations were completed in duplicate.

**TABLE 5:** List of fermentations conducted in the study and their abbreviations in text

Fermentation descriptions	Abbreviations
*L. thermotolerans* monoculture in duplicate	Lt
*S. cerevisiae* monoculture in duplicate	Sc
Co-culture with cell-cell contact in duplicate	CC+
Co-culture without cell-cell contact in duplicate	CC−

Co-culture fermentations excluding cell-cell contact (CC−) were achieved with the use of a compartmentalized bioreactor that can physically separate two microbial populations. This is done with the use of a membrane (0.1 µm cutoff), which was submerged into the fermentation medium inside one of the two compartments and connected to the adjacent compartment. With the use of filter-sterilized nitrogen gas applied into the headspace of one of the two compartments, medium was exchanged into the other compartment. The fermentation medium was continuously pumped from one compartment to the next until fermentations were completed ([total sugar] = <5 g/L). Experimental procedures were kept the same (except for removal of membrane) for co-cultures with cell-cell contact (CC+) and monoculture fermentations (Sc; Lt).

### RNA extraction, sequencing, and data processing

To avoid possible transcriptomic differences because of the physical presence of the membrane and the pumping, the entire media transfer system and the membranes were maintained for monocultures and the cell-cell contact co-cultures up until the last RNA sampling point. Hereafter, the membrane was removed where applicable. For RNA extractions, samples were taken at 2 h and 24 h. Total RNA extractions were performed using the hot phenol method (67). In total, two biological and two technical repeats of each fermentation type were sequenced. Monocultures (Lt and Sc) as well as CC− (CC−_Lt and CC−_Sc) samples were combined, respectively, and extracted together, therefore representing manually “mixed” monoculture (Lt/Sc) and CC− samples. Therefore, four mixed sample libraries (two biological and two technical repeats) of each fermentation type (Monoculture, CC+, and CC−) were created and sequenced. RNA concentrations and quality were determined using a Nanodrop 2000 Spectrophotometer (Thermo Fisher Scientific, Wilmington, DE, USA), and RNA integrity (RIN) was determined using a Bioanalyzer, 2100 expert software (Agilent, Santa Clara, CA, USA) and using the eukaryote total RNA program. Samples with a 260/280 (nm) ratio of greater than 2 and a RIN of 8 or more were sequenced. Library preparation and sequencing were performed by VIB Nucleomics core (KU, Leuven, Belgium) according to a previous study (28). After sequencing, adapters were trimmed at the end (at least 10 bp overlap and 90% match) using cutadapt 1.15 (68), and reads shorter than 35 bp were removed. Hereafter, low-quality ends (< Q20) were removed using FastX 0.0.14 (http://hannonlab.cshl.edu/fastx_toolkit/index.html), and again, reads shorter than 35 bp were removed. Lastly, poly-A-reads, ambiguous reads (containing N), low-quality reads (> 50% of the bases < Q25), and artifact reads were removed using FastX and ShortRead 1.36.1 (69). Broken pair reads and contaminants were removed using bowtie 2.3.3.1.

### Data analysis and identification of differentially expressed genes

Following the above pre-processing steps, reads were aligned to the reference genomes of *S. cerevisiae* S288C and *L. thermotolerans* CBS6340 using STAR 2.5.2b (70). Gene annotation was performed using a combined reference genome S288CplusLT to which all samples were aligned (28). Merging of the genomes proved successful, since a cross-mapping attempt between *S. cerevisiae* and *L. thermotolerans* reference genomes was found to be <1% (28). Reads with non-primary mappings or that have a mapping quality of ≤20 were removed using samtools 1.5 (71). Subsequent *bam* files were obtained and along with a corresponding feature file (concatenated *S. cerevisiae* S288C and *L. thermotolerans* CBS 6340 reference genomes in *gff* format) and were used to calculate the number of reads mapping to a specific gene using htseq-count 0.9.1 (72). Genes for which all samples had less than 1 count-per-million were removed (73). A full quantile normalization was performed using EDAseq 2.6.2 (74). This step accounts for differences in GC content and transcript length within samples and differences in library size and RNA composition between samples. Hereafter, data for technical repeats were combined, and biological repeats were represented by the calculated average. Lastly, edgeR 3.14.0 (31) was used to compare the remaining two biological repeats of each fermentation type by performing statistical analysis and identification of differentially expressed genes according to previous work (28). Six comparisons between fermentation types and species were made according to Table 6.

**TABLE 6:** Comparisons made for differential gene expression analysis for *S. cerevisiae* and *L. thermotolerans*

Species being analyzed	Comparisons	Description of comparisons
*S. cerevisiae*	1. CC+ VS Sc	*S. cerevisiae* differentially expressed genes in the CC+ fermentations when compared with monoculture
	2. CC− VS Sc	*S. cerevisiae* differentially expressed genes in the CC− fermentations when compared with monoculture
	3. CC+ VS CC-	*S. cerevisiae* differentially expressed genes in the CC+ fermentations when compared with CC-
*L. thermotolerans*	4. CC+ VS Lt	*L. thermotolerans* differentially expressed genes in the CC+ fermentations when compared with monoculture
	5. CC- VS Lt	*L. thermotolerans* differentially expressed genes in the CC- fermentations when compared to monoculture
	6. CC+ VS CC-	*L. thermotolerans* differentially expressed genes in the CC +fermentations when compared to CC-

For each comparison, differential expression was computed and expressed using the underlined settings as experimental, therefore, upregulated, or downregulated within the underlined fermentation type (Table 6). Data with log_2_ fold changes > 1 and an adjusted *P*-value < 0.05 were considered significant and analyzed further. VENN diagrams were constructed using VENNY 2.1.0 (75). A GO term enrichment analysis was performed using Amigo2 (76) and redundant GO terms were removed using REVIGO (77).

### qRT-PCR validation of RNA-seq data and further characterization of genes of interest

#### Fermentation setup

Additional to strains EC1118 and Y1240, *T. delbrueckii* strain BIODIVA (Lallemand Inc.), and *K. marxianus* strain IWBT Y885 obtained from SAGWRI (Stellenbosch University) were used. Yeast cultures were maintained at 4°C on YPD agar (20 g/L glucose, 20 g/L peptone, 10 g/L yeast extract, and 20 g/L agar). Fermentations were performed in the same synthetic grape must medium used in previous work (24). Four types of monoculture fermentations were performed (one for each species listed above) and were completed in triplicate. Three types of co-culture fermentations were performed: *S. cerevisiae* simultaneously inoculated with *L. thermotolerans*, *T. delbrueckii,* and *K. marxianus,* respectively, and were completed in triplicate. To obtain pre-cultures, a single colony was inoculated into 100 mL YPD broth and incubated overnight at 30°C and 100 rpm. Prior to inoculation, the pre-cultures were centrifuged at 4,000 × *g* and washed with 0.9% (m/vol) NaCl. Monocultures were inoculated at 2 × 10^6^ cells/mL, and co-cultures were inoculated at 1 × 10^6^ cells/mL of respective species (total of 2 × 10^6^ cells/mL). Fermentations were conducted in 200 mL of synthetic grape must in Erlenmeyer flasks and kept at 30°C on a shaker (100 rpm). Throughout fermentation, samples were taken at regular intervals, and growth was monitored on Wallerstein (WL) (Sigma-Aldrich, St. Louis, MO, USA) nutrient agar plates. Flasks were also weighed before and after each sampling to monitor fermentation progression.

#### qRT-PCR analysis

Qualitative real-time polymerase chain reaction was performed for some genes of interest (Table S1). RNA was extracted at 2 h, 12 h, and 24 h according to the same method used in the transcriptomic analysis, and gene expression in *S. cerevisiae*, *L. thermotolerans*, *T. delbrueckii,* and *K. marxianus* was quantified. To get rid of any DNA contamination, the samples were treated using RQ1 RNAse free DNase from Promega (Madison, WI, USA). The RNA was precipitated by adding 0.1 vol of a 3 M Sodium acetate solution (pH 5.2) and 2.5 volumes of 100% ethanol and left overnight at −20°C. Thereafter, the samples were centrifuged at maximum speed for 10 min at 4°C, the supernatant was removed, and the pellet resuspended was in 30 µL of ultrapure water treated with diethylpyrocarbonate (DEPC). The concentration and quality of samples were analyzed using a Nanodrop 2000 Spectrophotometer from Thermo Fisher Scientific (Waltham, MA, USA) and gel electrophoresis (1% agarose gel, ethidium bromide staining). To ensure the absence of DNA, PCR amplification using Ex Taq DNA polymerase (Takara Bio Inc., Kasatsu, Shiga, Japan) of the ITS1-5.8S rRNA-ITS2 gene was performed, and no amplicons were obtained (1% agarose gel, ethidium bromide staining), thus indicating successful DNase treatment of the RNA samples. The primer set used was ITS1 (5′-TCCGTAGGTGAACCTCGCG-3′) and ITS4 (5′-TCCTCCGCTTTATTGATATGC-3′). The PCR program was as follows: 95°C for 5 min; 30 cycles of 95°C for 30 s, 52°C for 30 s, 72°C for 1 min; 72°C for 10 min. cDNA was synthesized using the ImProm-IITM Reverse Transcription System from Promega (Madison, WI, USA) with Oligo(dT)15 primers.

A QuantStudio 3 Real-Time PCR system from Applied Biosystems with QuantStudio Design & Analysis Software v1.5.1 (Life Technologies, Carlsbad, CA, USA) was used to perform qRT-PCR. Reference genes for each yeast can be found in Table S1. Two reference genes were used for each qRT-PCR cycle, and the average CT value of the two was used in the ΔΔCT calculations. Primers were designed using the NCBI Primer design tool (https://www.ncbi.nlm.nih.gov/tools/primer-blast/) and were obtained from Inqaba biotec (Pretoria, South Africa). Genes were amplified using the Ampliqon RealQ plus 2x Master Mix Green (Ampliqon A/S, Odense, Denmark). Within each run, a negative control was included. PCR cycling conditions for primers (excluding Taqman) were as follows: hold stage of 50°C for 2 min and denaturation at 95°C for 10 min; 40 cycles of 95°C for 15 s and (annealing temperatures, Table S1) °C for 30 s and 72°C for 32 s; melt curve stage of 95°C for 15 s, 60°C for 1 min, and 95°C for 1 s. PCR cycling conditions for Taqman primers were as listed above excluding a melt curve stage. These customized Taqman hydrolysis probe sets were obtained from Thermo Fisher Scientific (Waltham, MA, USA). For each primer pair used, primer efficiency (E) was determined by making a serial dilution of cDNA, plotting C_T_ values (y-axis) VS log([cDNA]) (x-axis), calculating the slope of resulting C_T_ values and using the formula E = 100 × (10^−1/slope^ − 1).

A comparative critical threshold method (ΔΔC_T_) was used to calculate relative expression (RE) and log_2_ fold changes (78). Comparing monocultures (control) with co-cultures (experiment) poses an added challenge: an experiment group containing cDNA from two species. The Quantstudio 3 software cannot normalize for differences between cDNA composition; therefore, we added an additional step to the ΔΔC_T_ calculation to account for differences in cDNA concentration between the control and experiment groups (File S2). In the case of large transcriptional differences, this challenge does not pose a threat; however, in the case where genes are expressed at lower levels (regardless of treatment), small, meaningful differences may be missed if this is not accounted for.

Therefore, we adopted the following ΔΔC_T_ approach:

1. For the control (monocultures) and experiment (co-cultures):
  ΔC_T_ = C_T_ (target gene) – C_T_ (reference gene)
2. Added normalization step to account for differences in cDNA concentrations between control and experimental groups
  ΔC_T_norm (control) = ΔC_T_ + Calculated C_T_norm
3. ΔΔC_T_ = ΔC_T_ (experiment) - ΔC_T_norm (control)
4. RE (fold change) =2^−ΔΔCT^
5. Log_2_ fold change = Log_2_ (RE)

### Statistical analysis

qRT-PCR data were analyzed through analysis of variance (one-way ANOVA) and a least-significance-difference (LSD) test using Statistica software version 13.5.0.17 (StatSoft Inc., Tulsa, OK, USA). Differences were considered significant when *P*-values < 0,05. Relative gene expression graphs represent the average of three biological repeats, and error bars represent standard deviation. Data points that were statistically similar were annotated with an identical letter and data points that were statistically different were annotated with different letters.

## Data Availability

All supporting sequencing reads have been submitted to the National Center for Biotechnology Information (NCBI) Sequence Read Archive (SRA) database as raw FASTQ data files under the BioProject no. PRJNA902701. All raw FASTQ files and metadata can be located at the following link: https://www.ncbi.nlm.nih.gov/sra/PRJNA902701.

## SUPPLEMENTAL MATERIAL

File S1 spectrum.00572-23-s0001.xlsx Differentially expressed genes.

File S2 spectrum.00572-23-s0002.docx Normalization steps to account for differences in cDNA concentrations.

Figure S1 spectrum.00572-23-s0003.pdf Enriched GO-terms.

Figure S2 spectrum.00572-23-s0004.pdf Enriched GO terms for physical contact.

Table S1 spectrum.00572-23-s0005.docx Primers for Q-PCR.

ASM does not own the copyrights to Supplemental Material that may be linked to, or accessed through, an article. The authors have granted ASM a non-exclusive, world-wide license to publish the Supplemental Material files. Please contact the corresponding author directly for reuse.
