# Supplementary material for: Physical cell-cell contact elicits specific transcriptomic responses in wine yeast species
Source: Microbiol Spectr. 2024 Jul 16;12(8):e00572-23. doi: 10.1128/spectrum.00572-23 (PMC11302351; doi:10.1128/spectrum.00572-23)
Supplement: File S2 — Normalization steps to account for differences in cDNA concentrations. [file spectrum.00572-23-s0002.docx]

**Supplemental file S2: Normalization steps to account for differences in cDNA concentrations between monocultures and co-cultures**

Using *L. thermotolerans* and *S. cerevisiae* as an example, the following steps were taken in the case of each *S. cerevisiae* and non-*Saccharomyces* pairing:

Observing the plate counts at the time of RNA extraction in the three biological repeats, the ratios of each species were calculated in terms of percentages. See example in Fig. 1.

**FIG 1** Plate counts (CFU/mL) calculated for *L. thermotolerans* and *S. cerevisiae* in the Erlenmeyer CC+ fermentations expressed as percentages of the total population size for three biological repeats at 24 h.

When primer efficiencies were determined, serial dilutions were performed (either 2 × or 5 × dilutions) of co-culture cDNA and the resulting change in C_T_ value between each dilution was observed to be more or less 1 (2 ×) or 2.5 (5 ×). See example in Table 1.

**TABLE 1** Illustration of the increase in CT value of two *S. cerevisiae* qRT-PCR primers as co-culture cDNA was decreased per dilution series: 2 × (50 % reduction in cDNA per dilution) and 5 × (80 % reduction in cDNA per dilution). Standard deviation indicated as ±.

|  |  | *ALG9* | *TAF10* | |  |
| --- | --- | --- | --- | --- | --- |
|  | [cDNA] (ng) | Difference in CT value with each reduction | | | Average difference |
| 50% reduction in cDNA | 21.0 | - | | - | **+ 1.06** ± 0.24 |
|  | 10.5 | + 0.66 | | + 1.04 |  |
|  | 5.3 | + 1.40 | | + 0.92 |  |
|  | 2.6 | + 1.11 | | + 1.06 |  |
|  | 1.3 | + 0.94 | | + 1.37 |  |
| 80% reduction in cDNA | 40.0 | - | | - | **+ 2.37** ± 0.38 |
|  | 8.0 | + 1.99 | | + 2.00 |  |
|  | 1.6 | + 2.40 | | + 2.38 |  |
|  | 0.3 | + 2.50 | | + 3.01 |  |
|  | 0.1 | + 2.74 | | + 1.96 |  |

We constructed a scale predicting a theoretical increase in C_T_ value, should a specific dilution of cDNA be made (Table 2).

**TABLE 2** Estimated increase in CT value based on primer efficiency dilution series

| Times dilution | % Reduction in cDNA | Theoretical resulting increase in C_T_ value |
| --- | --- | --- |
| 2× | 50 | 1 |
| 3× | 67 | 1.5 |
| 4× | 75 | 2.0 |
| 5× | 80 | 2.5 |

Along with the calculated plate ratios for each *S. cerevisiae* and non-*Saccharomyces* yeast pairing, the scale was used to calculate the increase in C_T_ value of the monocultures needed in order for its [cDNA] to be similar to the respective co-cultures, for each species. See example in Table S3. In this case, *S. cerevisiae* and *L. thermotolerans* plate ratios were always close to 50 %, therefore, more or less 50 % reduction in [cDNA] (2 × dilution) would be needed in respective monocultures.

**TABLE 3** Calculation of increase in ∆C_T_ needed to account for the differences in [cDNA] between mono- and co-cultures for *S. cerevisiae* and *L. thermotolerans*

|  | Ratios of Co-Cultures (%) | Times dilution of monoculture needed to theoretically have equal [cDNA] to co-culture | Consequent increase in ∆C_T_ value needed in normalization step of calculations |
| --- | --- | --- | --- |
| Biological 1 Sc | 45.2 | 2.21 × | 1.107 |
| Biological 1 Lt | 54.8 | 1.82 × | 0.912 |
| Biological 2 Sc | 50.0 | 2 × | 1.000 |
| Biological 2 Lt | 50.0 | 2 × | 1.000 |
| Biological 3 Sc | 48.3 | 2.07 × | 1.036 |
| Biological 3 Lt | 51.7 | 1.93 × | 0.967 |
