# Supplementary material for: Physical cell-cell contact elicits specific transcriptomic responses in wine yeast species
Source: Microbiol Spectr. 2024 Jul 16;12(8):e00572-23. doi: 10.1128/spectrum.00572-23 (PMC11302351; doi:10.1128/spectrum.00572-23)
Supplement: Figure S1 — Enriched GO-terms. [file spectrum.00572-23-s0003.pdf]

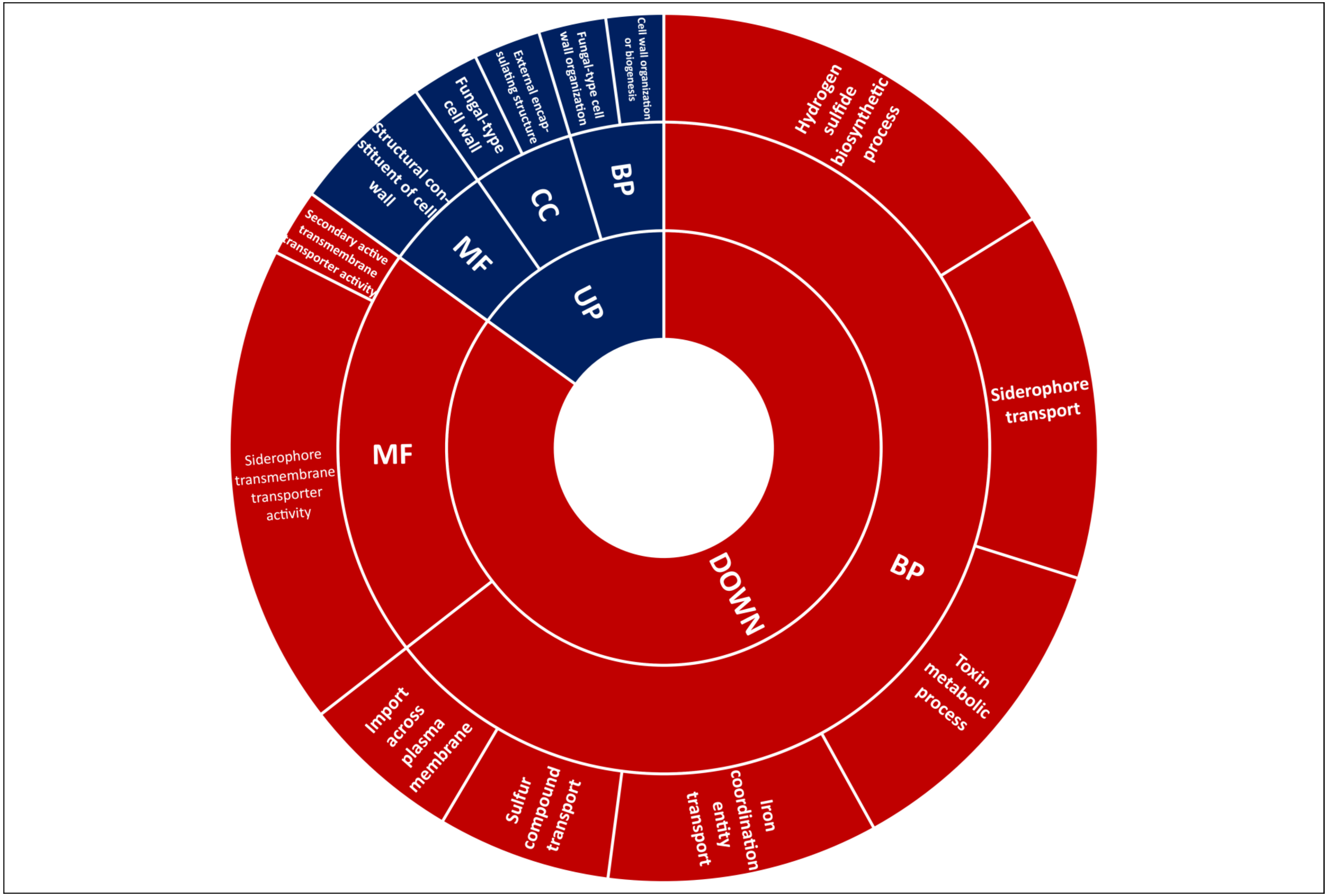

FIG S1 Sunburst chart representing shared enriched GO terms for genes that were differentially expressed between two physical contact comparisons (CC+_VS_CC-; CC+_VS_Sc) for *S. cerevisiae*. The size of blocks containing specific GO terms correlate to the average percentage of genes enriched in relation to total genes associated with specific GO terms for both comparisons. BP: Biological process; MF: Molecular function; CC: Cellular component; Up: Up-regulated; Down: Down-regulated.
