# Supplementary material for: Physical cell-cell contact elicits specific transcriptomic responses in wine yeast species
Source: Microbiol Spectr. 2024 Jul 16;12(8):e00572-23. doi: 10.1128/spectrum.00572-23 (PMC11302351; doi:10.1128/spectrum.00572-23)
Supplement: Figure S2 — Enriched GO terms for physical contact. [file spectrum.00572-23-s0004.pdf]

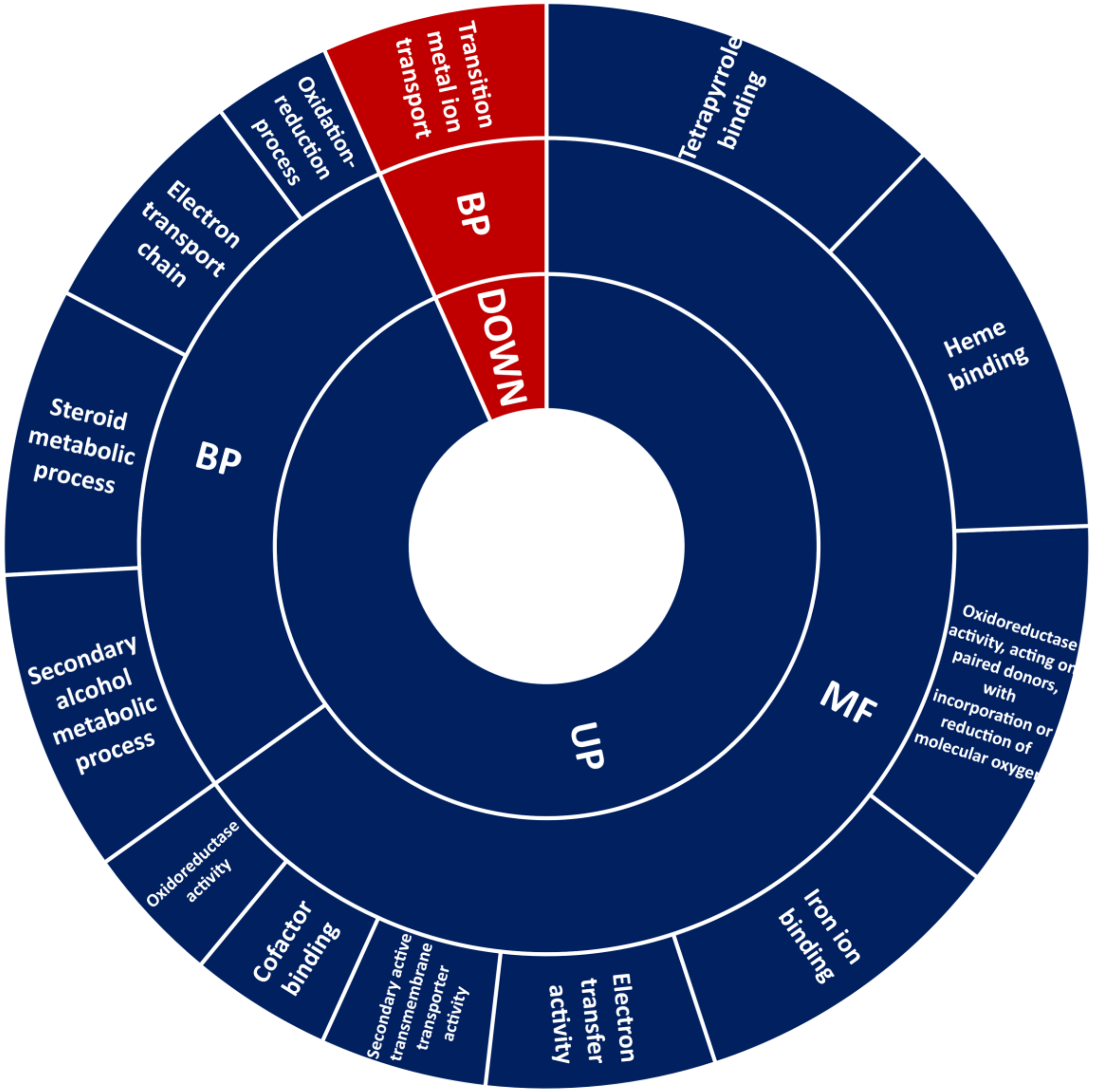

FIG S2 Sunburst chart representing shared enriched GO terms for genes that were differentially expressed between two physical contact comparisons (CC+_VS_CC-; CC+_VS_Lt) for *L. thermotolerans*. The size of blocks containing specific GO terms correlate to the average of percentage of genes enriched in relation to total genes associated with specific GO terms for both comparisons. BP: Biological process; MF: Molecular function; CC: Cellular component; Up: Up-regulated; Down: Down-regulated.
