# Supplementary material for: Physical cell-cell contact elicits specific transcriptomic responses in wine yeast species
Source: Microbiol Spectr. 2024 Jul 16;12(8):e00572-23. doi: 10.1128/spectrum.00572-23 (PMC11302351; doi:10.1128/spectrum.00572-23)
Supplement: Table S1 — Primers for Q-PCR. [file spectrum.00572-23-s0005.docx]

**TABLE S1** qRT-PCR primer pairs used to validate RNA-seq data and their corresponding sequences, amplicon sizes and annealing temperatures used

| Gene name and Locus tag on NCBI | Primer sequence (5’-3’) | Amplicon size (bp) | Annealing temperature used (°C) |
| --- | --- | --- | --- |
| Reference genes | | | |
| Sc: *TAF10*  *YDR167W* | F: CACCTTTTCCATCGGTTGCG  R: CCCATCATCCACTACAGCCTC | 110 | 60 |
| Sc: *ALG9*  *YNL219C* | F: ATTGGGGCGACGATGTCAAT  R: CGGTAAAGAGTGGCCGATCA | 57 | 60 |
| Lt: *TAF10*  *KLTH0G11352* | F: TCCCTGATCCGTTGTCAAGC  R: GAGGAAGCCCCAATCGACAA | 88 | 57/60*^a^* |
| Lt: *TEF1*  *KLTH0F11726* | F: GCCTCTTCCAAGGTCTTACCG  R: CAGGTCATTGTCTTGAACCACC | 143 | 57/60*^a^* |
| Td: *TAF10*  *TDEL0F04840* | F: TGCTGAGAAAAGCAGCGAGA  R: TCATCTAGACGTGGCTTCGC | 57 | 60 |
| Td: *ALG9*  *TDEL0A00670* | F: TCACGGGGCATGATATGTGG  R: TGGCCAGCAGATAATTGCGA | 92 | 60 |
| Km: *TAF10*  *KLMA_50525* | F: CCAGTGGATAACGAAGCACTCT  R: TGCTGCTTCTTCTTGCTCCA | 51 | 60 |
| Km: *ALG9*  *KLMA_50609* | F: CCATTTGGCCCATCTCAGGA  R: TGGTATAGACCCACTGCGGA | 130 | 60 |
| Genes of interest | | | |
| Sc: *TIR2*  *YOR010C* | F: TCGGAAACAGTGCTAGTGGC  R: AAAGCCTCCGCCATTTCTCA | 62 | 60 |
| Sc: *PAU5*  *YFL020C* | F: AGCGCTAGAGATAGCTGGCTT  R: TTGACTGGTATTCCCGCCG | 90 | 60 |
| Sc: *PAU17*  *YLL025W* | F: TTGAGACCAGCCATCTCCAG  R: AGCAGTGTAGATACCGTCTGC | 51 | 60 |
| Sc: *PAU23*  *YLR037C* | F: CAGTTGGATGAGCAGCTTGGA  R: CCCCAGCCACCACTACTT | 126 | 60 |
| Lt: *HSP42*  *KLTH0F09130* | F: GGGCCATGAAAGCCCTTAGT  R: ACTACGCCGAAAGACCACAG | 147 | 57 |
| Lt: *HSP78*  *KLTH0E02200* | F: GTTGCATGGTCTCGTGAAGC  R: GACGTCTGTCCGCCCTTC | 53 | 57 |
| Lt: *HSP104*  *KLTH0E06204* | F: GGAAGAGCCGGACCAGTTAG  R: GTCGTCGAGATCTTCGTCCC | 94 | 57 |
| Lt: *SSA2*  *KLTH0H08448* | F: GGTTGGTCCTTCAGCCTCAG  R: TATACCAGCAAGGTGGTGCC | 92 | 57 |
| Lt: *SSA3*  *KLTH0C06556* | F: TAGTCCACTTCCTCGACCGT  R: ATTCTACCAAGCTGGTGGCG | 150 | 57 |
| Lt: *FLO1*  *KLTH0E00242* | F: CCATGCAAATCAGTCGTGGG  R: CAGCAACAGCTTCACGGAAC | 102 | 60 |
| Lt: *FLO5*  *KLTH0C11924* | F: TGGCCACCACTGTTTTCACT  R: CCGAGTAGGTCGAGGTCTCA | 54 | 57 |
| Td: *HSP12*  *TDEL0C00880* | F: TGGTGAAAGTGTCAAGCCAGA  R: CCTTACCAGCTTCAGTGTAGGA | 53 | 60 |
| Td: *HSP42*  *TDEL0F04930* | F: GGGCACAACCTCCATACCTG  R: TTGTGGAGCCCCCAAAAGAG | 134 | 60 |
| Td: *HSP104*  *TDEL0F01650* | F: TCCAACTCCACATCGTCGTC  R: CACAGAACGCGGATCCGATA | 147 | 60 |
| Km: *HSP12*  *KLMA_20771* | F: TAAGCACAAGTTGGGCGAGG  R: CACCACCGTGAACTTGCTTG | 56 | 60 |
| Km: *HSP104*  *KLMA_40099* | F: GCTAGCTGCATTTGTCGAGC  R: GTCCTCACCAAGTGCCTGTT | 123 | 60 |
| Km: *FLO5*  *KLMA_10197* | F: GGTTGTGCTCCTAAGGCAGAT  R: TTGAAGCATCCTGGTGCAGT | 98 | 60 |
| Taqman probe sets for Sc *FLO* genes*^b^* | | | |
| Gene name and Locus tag on NCBI | **Taqman assay ID** | **Amplicon size (bp)** | **Annealing temperature used (°C)** |
| Sc: *FLO5*  *YHR211W* | Sc04131874_s1 | 93 | 60 |
| Sc: *FLO9*  *YAL063C* | Sc04098078_s1 | 72 | 60 |

*^a^*Lt reference gene annealing temperature of 57°C used when testing all genes of interest, except *FLO1* (annealing temperature of 60°C used)

*^b^*Taqman probe sets obtained from Thermo Fischer Scientific (Waltham, MA, USA)

Sc: *Saccharomyces cerevisiae*; Lt: *Lachancea thermotolerans*; Td: *Torulaspora delbrueckii*; Km: *Kluyveromyces marxianus*
